# Anchoring on COVID-19: A Case Report of Human Granulocytic Anaplasmosis Masquerading as COVID-19

**DOI:** 10.5811/cpcem.2021.4.51970

**Published:** 2021-05-25

**Authors:** Mark J. Stice, Charles A. Bruen, Kristi J.H. Grall

**Affiliations:** * HealthPartners Institute/Regions Hospital, Department of Emergency Medicine, Saint Paul, Minnesota; † HealthPartners Institute/Regions Hospital, Department of Critical Care, Saint Paul, Minnesota

**Keywords:** Human granulocytic anaplasmosis, COVID-19, critical care, case report

## Abstract

**Introduction:**

Human granulocytic anaplasmosis (HGA) is caused by *Anaplasma phagocytophilum* and transmitted through the deer tick. Most cases are mild and can be managed as an outpatient, but rare cases can produce severe symptoms.

**Case Report:**

A 43-year-old male presented with severe respiratory distress mimicking coronavirus disease 2019 (COVID-19). Labs and imaging were consistent with COVID-19; however, polymerase chain reaction was negative twice. Peripheral smear revealed inclusion bodies consistent with HGA.

**Conclusion:**

Human granulocytic anaplasmosis is an uncommon diagnosis and rarely causes severe disease. Recognition of unique presentations can aid in quicker diagnosis, especially when mimicking presentations frequently seen during the COVID-19 pandemic.

## INTRODUCTION

Human granulocytic anaplasmosis (HGA) is a disease caused by *Anaplasma phagocytophilum* through the deer tick (*Ixodes scapularis*) as a vector.^1^ The majority of cases occur in the Midwest and Northeast United States,^2^ producing mild and nonspecific symptoms that can generally be managed as an outpatient. However, rare cases can cause severe illness necessitating inpatient and even intensive care unit (ICU) management.^3^,^4^ While most cases are contained to specific geographic regions, severe cases are uncommon. The nonspecific nature of symptoms can make diagnosis challenging, especially when presentations may mimic coronavirus disease 2019 (COVID-19) infection during a global pandemic.^4^

## CASE REPORT

A 43-year-old male arrived via emergency medical services as a transfer from a stand-alone emergency department (ED) with hypoxemia and severe respiratory distress. He provided a limited history secondary to his respiratory distress but noted he had experienced progressively worsening shortness of breath and chest pain over the prior 1–2 days. The patient had a COVID-19 exposure at an airport, approximately 7–10 days prior to arrival. Paramedics noted he had oxygen saturations in the low 80s on room air and improved to the mid 90s with 15 liters per minute (LPM) through a non-rebreather mask. He felt better with the supplemental oxygen but was still experiencing shortness of breath and speaking in two- to three-word sentences. He denied significant medical history other than tobacco abuse with recent cessation.

The patient’s initial vitals were notable for a temperature of 101.2^o^F, respiratory rate of 51 breaths per minute, and heart rate of 138 beats per minute. On examination, he remained in respiratory distress with profound tachypnea, but auscultation revealed clear bilateral lung sounds without wheezes, rhonchi, or rales. Other notable exam findings were diffuse patches of capillary dilation with each collection originating from a single locus scattered throughout the distal extremities, tachycardia with regular rhythm, and mild scleral icterus. After examination, the patient was transitioned to high-flow nasal cannula, and his work of breathing improved with a flow rate of 45 LPM and 100% oxygen. Consideration was given to initiating bilevel positive airway pressure (BiPAP); however, because his clinical presentation was suspicious for COVID-19 infection BiPAP was not started to reduce potential staff exposure. The patient intermittently self-proned with some subjective symptomatic improvement.

The patient’s notable laboratory work-up is described in the Table. A chest radiograph obtained showed bilateral interstitial and airspace infiltrates. In addition, the patient’s COVID-19 polymerase chain reaction (PCR) resulted negative.

Despite the negative COVID-19 polymerase chain reaction test (PCR), his clinical presentation and work-up appeared most consistent with severe COVID-19 pneumonia. Blood cultures were drawn, and the patient was started on cefepime and vancomycin in the event his presentation was a result of a bacterial pneumonia. A computed tomography (CT) angiography of the chest was obtained due to the elevated D-dimer and respiratory distress. No pulmonary embolism was present, but the CT did reveal extensive bilateral infiltrates consistent with a COVID-19 pneumonia (Image). After results had been obtained, a report from the transferring ED contained similar lab results, including a negative COVID-19 PCR. The patient was then admitted to the ICU for ongoing care.

**CPC-EM Capsule**

What do we already know about this clinical entity?

*Tick-borne infections are typically mild, but occasionally have severe manifestations. The rarity of these presentations can make diagnosis challenging*.

What makes this presentation of disease reportable?

*This rare presentation of human granulocytic anaplasmosis closely mimicked the commonly seen coronavirus disease 2019 during the global pandemic*.

What is the major learning point?

*When an uncommon disease presents similarly to a common diagnosis, it is possible for anchoring bias to occur*.

How might this improve emergency medicine practice?

*Recognizing the possibility of anchoring bias can produce a faster, accurate diagnosis and lessen potential morbidity*.

After the patient transferred to the ICU, a third COVID-19 PCR test was obtained and resulted negative. The profound thrombocytopenia triggered a review of the patient’s hematopathology. His peripheral smear demonstrated neutrophil intracellular organismal inclusions consistent with HGA. Confirmatory anaplasmosis PCR testing was sent and would later result positive. The patient was empirically switched to doxycycline 100 milligrams (mg) twice a day antibiotic therapy for 14 days. Further information was later obtained from the patient’s wife, who noted the patient had been hunting approximately 1–2 weeks prior to presentation and had removed numerous ticks from himself afterward.

Despite treatment with doxycycline, the patient’s respiratory status worsened and he required intubation for acute respiratory distress syndrome on hospital day (HD) one and subsequent proning. The patient required five days of mechanical ventilation; he was then extubated and transferred out of the ICU on HD six. He had a progressive, severe, non-oliguric acute kidney injury thought to be secondary to acute tubular necrosis with creatinine peaking at 7.31 mg per deciliter on HD four, but never requiring hemodialysis. The patient’s thrombocytopenia required a platelet transfusion on HD zero and again on HD three after platelets dropped to 9 ×10^9^ per liter (10^9^/L). However, platelets improved to 183×10^9^/L by the time of ICU transfer and did not require further transfusions. The patient recovered and was discharged to home with home physical therapy after a 12-day hospitalization.

## DISCUSSION

Human granulocytic anaplasmosis is a disease caused by the gram-negative bacterium, *Anaplasma phagocytophilum*.^1^ It is transmitted by the deer tick (*Ixodes scapularis*), which is also the vector for Lyme disease and babesiosis.^2^ Although it has long been considered in veterinary pathology, HGA was not known to infect humans until it was discovered in 1994.^1^ Highest incidence in the United States occurs in regions endemic for the deer tick, which include the upper Midwest and Northeast regions, with 10 states recording over 90% of reported cases (Minnesota, Wisconsin, Massachusetts, New York, Maine, Connecticut, New Jersey, Rhode Island, Vermont, and New Hampshire), making the amount of time spent in an endemic area the highest risk factor.^2^,^3^ Most infections occur seasonally, corresponding with tick activity.^3^

Most patients are initially diagnosed with a mild viral illness that readily resolves with supportive care.^4^ Symptoms may include fevers, myalgias, headache, and rigors. Rash is surprisingly uncommon.^1^,^2^ Although most patients relate tick exposure approximately 1–2 weeks prior to onset of symptoms, one quarter of patients do not recall a tick bite.^4^ Lab testing often reveals leukopenia with a left shift, thrombocytopenia, and mild to moderate elevation in liver function testing.^4^

Thirty six percent of patients may develop more severe illness and require hospitalization, and 17% of these end up in the ICU.^4^ Life-threatening complications develop in 3% and include acute respiratory distress syndrome, acute renal failure, and hemodynamic collapse.^2^ Poorer prognosis is seen in elderly, immunocompromised patients, those with underlying malignancy, or in patients where there is a significant delay to diagnosis.^3^,^4^ While HGA can be quite serious, reported mortality rate is less than 1%.^3^,^4^ Diagnosis is via serologic testing for antibodies, although treatment with doxycycline may be started with presumptive diagnosis in patients who have fever, myalgias, and suggestive changes in laboratory values.^2^,^3^,^4^ Patients treated with doxycycline usually resolve symptoms within 48–72 hours.^4^

Coronavirus disease 2019, the viral illness that originated in Wuhan, China, and caused a global pandemic, has become readily recognized in EDs across the world.^5^,^6^ Typically patients present 4–8 days after exposure,^5^ describing fever, shortness of breath, cough, myalgias, fatigue, loss of taste and/or smell, or diarrhea.^7^ Several unusual phenomenon have been observed on examination, including the following: “happy hypoxemia” where patients are significantly hypoxemic without respiratory distress or perceived shortness of breath^8^; and “COVID toes” where patients exhibit purple discoloration in their distal toes, although some controversy exists as to whether this is specific to COVID-19 or generalizable to systemic inflammatory disease.^9^ Laboratory studies often show leukopenia, elevated liver transaminases, C-reactive protein, and D-dimer.^5^ Chest radiographs in COVID-19 patients frequently show bilateral involvement with mixed airspace and interstitial opacification.^10^ Chest computed tomography (CT) provides greater detail, and in severe disease ground-glass opacities can be observed in 100% of patients with a multilobular and bilateral distribution favoring posterior involvement.^11^

In our patient found to have HGA, his presentation mimicked numerous severe COVID-19 cases we have seen in our ED. Many aspects of his history and work-up were further consistent with our initial suspicion. The patient’s incubation period mirrored the duration described earlier, and his symptoms were consistent with those reported by the US Centers for Disease Control and Prevention. Although some controversy exists, the discoloration in his distal extremities appeared consistent with described “COVID toes.” Many laboratory results were suggestive of COVID-19 with elevated transaminases, C-reactive protein, D-dimer, and lymphocytopenia. Imaging studies matched those frequently seen in COVID-19 patients. Further confounding the diagnosis was his relief with self-proning, which has been a method used to reduce intubation and mechanical ventilation in COVID-19 patients by increasing oxygenation through recruitment of collapsed alveoli.^12^ While all of this was suggestive of COVID-19, his thrombocytopenia, hyponatremia, and two negative COVID-19 PCR tests were inconsistent with a COVID-19 infection. A single PCR test cannot completely rule out infection with sensitivities reported between 60–70%,^13^ and studies have shown CT findings can precede a positive PCR.^14^ However, after receiving the second negative COVID-19 PCR in the ED, our leading diagnosis needed to be reconsidered.

Although our ED is located in an endemic HGA area, his diagnosis was further challenging because other tick-borne illnesses are more prevalent, his presentation was outside of the typical infectious season, his illness severity was rare for HGA, and we did not have reports of tick exposure. Our failure to move beyond COVID-19 as the leading diagnosis represents anchoring bias, and it is commonly encountered in medicine.^15^ Bias arises from heuristics and mental shortcuts developed from experience to rapidly assess and treat patients; specifically, anchoring occurs when a physician locks on to a diagnosis despite the presentation of contrarian evidence.^15^ If a correct diagnosis had not been suggested by pathology assessing evidence while blinded to clinical context, the patient could have suffered significant morbidity or mortality by not receiving appropriate treatment.

## CONCLUSION

Human granulocytic anaplasmosis rarely presents with severe disease manifestations, even in endemic areas. A difficult diagnosis can be confounded when presentation mimics one frequently observed during the COVID-19 pandemic. Anchoring bias arises from heuristics used in medicine, especially in a clinical setting, necessitating rapid assessment and treatment such as in emergency medicine. Despite the need to provide expedited care, when examination and testing do not fit a diagnosis the situation requires reassessment. The case described above illustrates the importance of keeping a broad differential and discarding a diagnosis when it is not supported by evidence. Providing timely diagnosis and treatment is vital, but just as important is recognizing when anchoring bias may be present and then having the humility to admit you were wrong.

## Figures and Tables

**Image:**
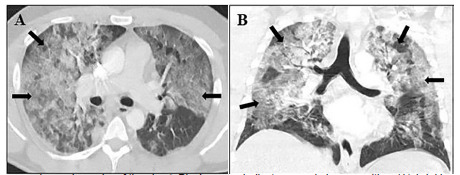
Computed tomography angiography of the chest. Black arrows indicate ground glass opacities. (A) Axial image showing bilateral central and peripheral ground-glass opacities. (B) Coronal image demonstrating bilateral ground-glass opacities in upper and lower lung fields.

**Table:** Patient’s significant laboratory values with site’s reference ranges.

Lab test (units)	Patient’s results	Reference range
Platelets (10^9^/L)	9	150–450
Lymphocytes (10^9^/L)	0.3	1.0–4.8
Sodium (mmol/L)	121	136–145
Creatinine (mg/dL)	2.16	0.73–1.18
AST (U/L)	644	10–40
ALT (U/L)	144	0–55
D-dimer (ug/mL)	>20.00	<0.50
C-reactive protein (mg/dL)	29.4	0.0–0.7
Procalcitonin (ng/mL)	16.67	<0.24

*L*, liter; *mmol*, millimoles; *mg*, milligrams, *dL*, deciliter; *AST*, aspartate transaminase; *U*, units; *ALT*, alanine transaminase; *ug*, micrograms; *mL*, milliliter; *ng*, nanograms.
